# Does pattern mixture modelling reduce bias due to informative attrition compared to fitting a mixed effects model to the available cases or data imputed using multiple imputation?: a simulation study

**DOI:** 10.1186/s12874-018-0548-0

**Published:** 2018-08-29

**Authors:** Catherine A. Welch, Séverine Sabia, Eric Brunner, Mika Kivimäki, Martin J. Shipley

**Affiliations:** 1 Department of Epidemiology and Public Health, University College London, Gower Street, London, WC1E 7HB UK; 2 INSERM U1018, Centre for Research in Epidemiology and Population Health, Düsternbrooker Weg, 20 Villejuif, France

**Keywords:** Multiple imputation, Informative attrition, Pattern mixture modelling, Longitudinal observational data

## Abstract

**Background:**

Informative attrition occurs when the reason participants drop out from a study is associated with the study outcome. Analysing data with informative attrition can bias longitudinal study inferences. Approaches exist to reduce bias when analysing longitudinal data with monotone missingness (once participants drop out they do not return). However, findings may differ when using these approaches to analyse longitudinal data with non-monotone missingness.

**Methods:**

Different approaches to reduce bias due to informative attrition in non-monotone longitudinal data were compared. To achieve this aim, we simulated data from a Whitehall II cohort epidemiological study, which used the slope coefficients from a linear mixed effects model to investigate the association between smoking status at baseline and subsequent decline in cognition scores. Participants with lower cognitive scores were thought to be more likely to drop out. By using a simulation study, a range of scenarios using distributions of variables which exist in real data were compared.

Informative attrition that would introduce a known bias to the simulated data was specified and the estimates from a mixed effects model with random intercept and slopes when fitted to: available cases; data imputed using multiple imputation (MI); imputed data adjusted using pattern mixture modelling (PMM) were compared. The two-fold fully conditional specification MI approach, previously validated for non-monotone longitudinal data under ignorable missing data assumption, was used. However, MI may not reduce bias because informative attrition is non-ignorable missing. Therefore, PMM was applied to reduce the bias, usually unknown, by adjusting the values imputed with MI by a fixed value equal to the introduced bias.

**Results:**

With highly correlated repeated outcome measures, the slope coefficients from a mixed effects model were found to have least bias when fitted to available cases. However, for moderately correlated outcome measurements, the slope coefficients from fitting a mixed effects model to data adjusted using PMM were least biased but still underestimated the true coefficients.

**Conclusions:**

PMM may potentially reduce bias in studies analysing longitudinal data with suspected informative attrition and moderately correlated repeated outcome measurements. Including additional auxiliary variables in the imputation model may also reduce any remaining bias.

**Electronic supplementary material:**

The online version of this article (10.1186/s12874-018-0548-0) contains supplementary material, which is available to authorized users.

## Background

Informative attrition is a potential source of bias in longitudinal data analysis, which occurs when participants drop out of a study and the reason for drop out is associated with the study outcome [1]. Analysing longitudinal data ignoring informative attrition may bias findings due to selection bias. For example, if lower cognitive functioning, the outcome, is associated with drop out, participants with lower cognitive function are more likely to be missing. Informative attrition can only be assumed to exist from our knowledge of the data; but this association cannot be determined since the required data are missing.

A few different approaches can reduce bias due to missing data in longitudinal studies. Firstly, fitting a mixed effects model (with random intercept and slope) to the available cases (AC) will exclude participants with missing outcome or exposure values at all data collection phases but will analyse participants who have values missing only at some phases by allowing for the missing data using the within and between participant correlations. However, the mixed effects model may not reduce all the bias due to informative attrition if not enough information exists in the data.

The second approach for handling missing data is multiple imputation (MI) [2]. MI repeatedly selects random values from the missing data distribution, given the observed data, defined using an imputation model. The repeated draws generate many imputed datasets and the mixed effects model is fitted to each dataset separately and these results combined using Rubin's rules [3]. An approach, validated to impute missing values in non-monotone, longitudinal data is the two-fold fully conditional specification (FCS) algorithm, which imputes missing values at each phase sequentially, conditional on the observed information at adjacent phases [4, 5]. One benefit of MI compared to the AC analysis is that MI can use additional information, as auxiliary variables, in the imputation model to reduce bias. However, to achieve unbiased results from imputed data, the data needs to have a plausible ignorable missingness mechanism, that is, the probability of the data being missing is not associated with the missing values, conditional on the observed data [2]. If informative attrition is present, the missingness mechanism is non-ignorable and MI alone cannot completely reduce the bias.

A third approach is pattern mixture modelling (PMM) [3], which can be used as a sensitivity analysis if the missingness mechanism is thought to be non-ignorable. The procedure first assumes an ignorable missingness mechanism and uses MI to impute missing values, generating multiple datasets. Next, to use the PMM approach, adjust the imputed values by a fixed value. Larger adjustment values suggest greater ignorable missing assumption violation. Finally, the mixed effects model is fitted using these adjusted values in each dataset and the results combined using Rubin's rules. Other sensitivity analyses exist, for example selection modelling, which specifies a selection distribution for those who drop out [3] or inverse probability weighting [6] which can correct for the bias due to missing data. However, we will focus on PMM since we can begin by assuming an ignorable missingness mechanism and then incorporate non-ignorable assumptions into the model.

Many clinical trial studies with longitudinal data recommend using PMM as a sensitivity analysis [7–10]. In general, clinical trial data have a monotone missingness pattern (non-response at a given phase will be missing at all later phases), which simplifies MI and, therefore, PMM. However, in observational longitudinal studies, data are often missing due to non-response as well as attrition, giving the data a non-monotone missingness pattern. In addition, the missingness mechanism for participants with repeated non-response status, who have not officially withdrawn from the study, may be more similar to participants with attrition status compared to participants who alternate between response and non-response. In this context, using MI to impute missing values may be more complex compared to clinical trials.

For this analysis, a simulation study was designed to evaluate these different approaches by comparing PMM to an available case mixed effects model and multiple imputation. Fully observed datasets, with known distributions and associations are generated, and a mixed effects model fitted to these datasets to obtain ‘true’ coefficients and standard errors (SE). Then, informative attrition is defined in the dataset using a non-ignorable missingness mechanism of our choice.

By replacing selected values with missing values, each approach can be used to account for bias due to informative attrition by analysing the data and comparing the coefficients and SE to ‘true’ estimates to assess bias and precision [11]. For this simulation study, we used distributions and associations in the Whitehall II study [12]. Data were first collected for over 10,000 civil servants in 1985 and data collection phases were repeated every 2-3 years. Participants completed a health and lifestyle questionnaire and, at alternate phases, attended a screening clinic. Over 30 years, analyses of the Whitehall II study have resulted in many publications. One investigated the association between smoking status at baseline (Phase 5) and 10-year cognitive decline using a mixed effects model with random intercept and slope [13]. This analysis was used as the basis for our simulation study to investigate whether informative attrition of participants with reduced cognitive function, who may have been unable to continue participation in the study, could give rise to bias in the estimates of association.

The aim of our study was to compare bias and precision of fitting the mixed effects model to the AC with an analysis which imputes data using the two-fold FCS algorithm and uses PMM to reduce bias due to informative attrition. With informative attrition, we expect least biased results when we apply methods which assume non-ignorable missingness such as PMM. However, by using a simulation study, we can assess whether the results are as expected and also quantify the difference in bias for PMM compared to approaches which assume ignorable missingness. In addition, simulation allows the effects of different percentages of informative attrition and different types of covariates with missing data to be assessed.

For our study, 1,000 fully observed datasets were simulated, each with 10,000 participants, having the same distributions and associations as observed in the Whitehall II study. From the missing data distributions observed in the Whitehall II study, an ignorable missingness mechanism for participants without attrition status and a non-ignorable missingness mechanism for participants with attrition status were first created. As it is not known how many with non-response status have a non-ignorable missingness mechanism, the analysis was repeated, generating a non-ignorable missingness mechanism for all participants with attrition or non-response status. We used a sensitivity analysis to investigate how results change if missing values are imputed for the time-independent covariate education at Phase 5 instead of time-dependent covariate smoking status.

## Methods

### Study design

Longitudinal records exist for *i = 1,...,N* independent participants with *Y*_*i,t*_ the outcome values for participant *i* at phase *t* (a time period when data collection occurs). It is assumed that explanatory variable *X* exists with values at *t = 1,...,T* (typically equally spaced) phases. Let *X*_*i,t*_ denote the value of variable *X* for individual *i* at phase *t*.

The substantive model (model of interest) is a linear mixed effects model adjusted for the explanatory variables' main effect and their interaction with data collection phase, together with random intercept *β*_*0i*_ and slope *β*_*1i*_:

1 $$ {Y}_{i,t}={\beta}_0+{\beta}_1{X}_{i,t}+{\beta}_2t\kern0.1em {X}_{i,t}+{\beta}_{0i}+{\beta}_{1i}\kern0.1em t+{\varepsilon}_{i,t} $$

### The two-fold fully conditional specification algorithm

Historically, to impute missing data for more than one variable, random draws were selected from a multivariate normal conditional distribution for the variables with missing values, conditional on the observed data, to obtain a complete dataset of observed and imputed values [14]. The approach generated multiple datasets, each analysed separately and the results combined using Rubin's rules [2]. In many cases, the multivariate normal model is difficult to define, for example if the rows or columns are ordered (such as with longitudinal data), or are not multivariate normally distributed, for example with different variable types (such as categorical). A more flexible approach, fully conditional specification (FCS) [15], selects random draws from separate conditional, univariate imputation models for each variable with missing data, repeatedly cycling through each variable in turn. Compared to fitting a multivariate normal model, FCS is computationally convenient and, despite a lack of theoretical justification, simulation studies found using FCS to impute missing values achieves similar results compared to using a multivariate model [16, 17].

For longitudinal data with *t = 1,...,T* phases and *j = 1,...,J* variables measured at each phase, FCS imputes missing values for the *J* variables at each phase *t*. FCS repeats these imputations at each phase and the *J T* imputations constitute one iteration. However, the imputed data may lose the correlation structure between phases and biased estimates may be observed from analysing data imputed using FCS by not conditioning on measurements at other phases. The *J T* variables could be imputed simultaneously but, with many highly correlated repeated measurements, this may cause convergence problems due to collinearity, particularly for categorical variables [18].

Collinearity issues can be avoided and the correlation structure maintained in the longitudinal data by imputing using the two-fold FCS algorithm [4], which imputes missing values at phase *t* using FCS conditional on values at phase *t* and adjacent phases; a within-time iteration. The two-fold FCS algorithm repeats *b*_*W*_ within-time iterations at each phase, generally in time order, and completes one among-time iteration when all phases are imputed. This is repeated for *b*_*A*_ among-time iterations. Once the specified within-time and among-time iterations are complete, the first imputed dataset consists of current imputed and observed values. This is repeated *M* times to create *M* imputed datasets, which are each analysed separately and the results combined using Rubin's rules [2]. By conditioning on only adjacent phases, the two-fold FCS algorithm is more efficient compared to approaches which do not use information at other phases and can impute missing values in large datasets with many participants, phases and variables [5].

Missing outcome (*Y*) and covariate (*X*) values were imputed using the two-fold FCS algorithm, each imputed dataset analysed using substantive model (Eq. 1) and the results combined using Rubin's rules. The imputation model included all variables specified in Eq. 1, including the outcome, but no additional auxiliary variables were included to simplify the interpretation of the results. No interactions with time were specified since the two-fold FCS algorithm includes interactions with time by imputing each time point separately. The two-fold FCS algorithm was used, with 5 within-time iterations and 20 among-time iterations, to impute missing values at Phases 3, 5, 7, 9 and 11. Smoking status was conditioned on, at baseline only (no other phases), when smoking status was not missing, in order to avoid convergence issues due to collinearity. Twenty imputed datasets were generated, the substantive model fitted to each dataset, and the results combined using Rubin's rules.

### Pattern mixture modelling

Analysing data imputed using the two-fold FCS algorithm can achieve unbiased findings if an ignorable missingness mechanism can be assumed for the data. However, in longitudinal observational studies, attrition is often associated with the missing outcome values *Y* (informative) and non-ignorable missingness [19]. If an ignorable missingness mechanism cannot be assumed, fitting a mixed effects model (Eq. 1) to the AC or data imputed using the two-fold FCS algorithm may still produce biased results. An approach that reduces the bias due to informative attrition is required.

In this situation, the outcome distributions may differ, depending on the missing data patterns. For example, there may be different patterns of missing observations, each potentially with a different joint distribution of partially observed and fully observed data with the overall density being the average of these patterns [3]. For each pattern, the joint distribution of the partially and fully observed variables is specified, which implies, within each pattern, a conditional distribution exists for the partially observed data given the fully observed data. To apply PMM, an ignorable missingness is assumed initially and missing values imputed using the two-fold FCS algorithm. These imputed values are then changed to reflect explicit assumptions about the difference between the observed and conditional distribution when the variables are unobserved [3].

In our data we have two missing data patterns, observed outcomes and missing outcomes. The distribution of the observed outcome pattern is given by Eq. 1. For the missing outcome pattern, we define an attrition indicator *R*_*i,t*_ for participant *i* who leaves the study at phase *t* and add this to the observed outcome pattern:

2 $$ {Y}_{i,t}={\beta}_0+{\beta}_1{X}_{i,t}+{\beta}_2t\kern0.1em {X}_{i,t}+{\beta}_{0i}+{\beta}_{1i}\kern0.1em t+{\varepsilon}_{i,t}+{kR}_{i,t} $$

where *k* is the assumed mean difference between the imputed outcome distribution and the unknown true distribution which cannot be estimated from the observed data. If *k = 0*, the missingness mechanism is ignorable, otherwise for *k ≠ 0* the mechanism is non-ignorable. Larger *k* suggests a greater violation of the ignorability assumption.

The PMM steps are; first, use the two-fold FCS algorithm to impute the missing data and generate *M* imputed datasets. For each imputed dataset, change the already imputed outcome values *Y*_*i,t*_, missing due to attrition *R*_*i,t*_, by *k*. Finally, fit the substantive model (Eq. 1) to the imputed dataset with updated outcome values.

### Data generation and simulation process

A simulation study was designed using the Whitehall II study data. Exposure-outcome relationships were simulated using an existing epidemiological investigation of the association between smoking status at baseline (Phase 5) and 10-year cognitive decline using cognitive function measured at Phases 5, 7 and 9, each 5 years apart [13]. In the original study, Sabia, et al., stratified by sex and derived a 4 category smoking status variable. To simplify the analysis for the simulation study, only male participants and 3 smoking status categories (current smokers, ex-smokers and never smokers) were used. The distribution and associations of the variables and missing data were replicated.

For each of the 1,000 simulations, the following steps were used and are described in detail later in this section:

1. Generate samples of *N* = 10,000 male participants.
2. Fit substantive model to simulated data (with no missing values), record parameter estimates and SE.
3. Replace outcome and explanatory variable values with missing values:

- if not missing due to attrition - change observations to missing using an ignorable missingness mechanism at each phase.
- if missing due to attrition - change observations to missing using a non-ignorable missingness mechanism at each phase.

4. Fit substantive model to AC, record parameter coefficients and SE.
5. Impute missing data using the two-fold FCS algorithm and fit the model of interest to each imputed dataset, combine the results using Rubin’s rules [2] and record the imputation-based parameter coefficients and SE.
6. Apply PMM to the imputed datasets from step 5, adjust imputed values by a fixed value, re-analyse and record the imputation-based parameter coefficients and SE.

### Data generation mechanism

We generated data at Phases 3, 5, 7, 9 and 11 because smoking status and cognitive function were recorded at these clinic phases. The substantive model was fitted to data collected at Phases 5, 7 and 9 but we also generate data at Phases 3 and 11 to inform the imputation of missing values at the phases in between. We generated the following time-independent categorical variables at baseline: age in years (5 categories); and socioeconomic status measured using occupational grade (high [administrative], intermediate [professional or executive] and low [clerical or support]) and education (primary school [until age 11 years], secondary school [until age 18 years] or university). Finally, we generated time-dependent categorical smoking status (current smoker, ex-smoker and never smoker).

Cognitive function was assessed using 5 tests.

1. Short term verbal memory - 20 one- or two-syllable words presented at 2 sec intervals that the participants had 2 min to recall in writing.
2. Vocabulary - Mill Hill Vocabulary Test [20] in its multiple-choice format consisting of a list of 33 stimulus words ordered by increasing difficulty and 6 response choices.
3. Reasoning - Alice Heim 4-I test, total verbal and mathematical reasoning tasks completed in 10 min (out of 65) [21].
4. Phonemic fluency - total words beginning with ‘S’ recalled verbally in 1 min [22].
5. Semantic fluency - total animals recalled verbally in 1 min [22].

A global cognitive score using all 5 cognitive function tests was created to minimize problems due to measurement error [23, 24]. The scores on each test for the entire cohort were standardised to z scores (mean [SD] = 0 [1]) using the mean and standard deviation at Phase 5 (baseline). To calculate the global cognitive function, the z scores were averaged to create a global cognitive score and standardised again using the mean and standard deviation at Phase 5.

We compared results for two time-dependent outcome measures with different size correlations among repeated measurements; standardised memory score (correlations 0.45) and standardised global score (correlations 0.97). Each outcome was generated using two different mixed effects models with random intercept and slope fitted to data collected at Phases 5, 7 and 9, conditional on variable measurements at baseline (Phase 5): smoking status, age, occupational position and education. The models also included an interaction between each variable and time.

The data generation details are described in the Additional file 1: Appendix.

### Parameters used for data generation

To derive the model parameters used for each data generation step, the mixed effects models were fitted to data from the cohort of Whitehall II study participants to obtain coefficients, considered to be the ‘true’ estimates in the simulation study. Any phases with missing smoking status were replaced with ‘never smoker’ if participants only had ‘never smoker’ smoking status recorded and, otherwise, were imputed as either ‘current smokers’ or ‘ex-smokers’. Welch, *et al*., found using this approach reduced the missing data, ensured consistent smoking status recording and simplified MI using the two-fold FCS algorithm [5]. Any male participants who died or withdrew from the study before Phase 5 or those with missing cognitive function score or smoking status at Phases 5, 7 or 9 were excluded.

### Missingness mechanism

Two different missingness mechanisms were investigated to compare results from imputing missing values for time-independent and time-dependent covariates. For the first missingness mechanism, a fixed percentage of the cognitive function measures (outcome) and smoking status (exposure) at each phase were changed to missing. For the second missingness mechanism, a percentage of the cognitive function measures (outcome) at each phase and education at baseline (covariate) were changed to missing. For these variables, the percentage of values changed to missing was similar to the percentage missing observed in the Whitehall II study.

One of the following participation statuses was generated for each participant at each phase:

- Response - participated at a given phase, but may have missing values for some variables (item non-response).
- Non-response - does not participate at a given phase so all variables have missing values (unit non-response).
- Death - before phase, confirmed by death certificate.
- Attrition - informed Whitehall II study they no longer wish to participate before the phase.

At Phases 3 and 5, only response or non-response status levels were generated, since all participants who died or dropped out before phase 5 were excluded. All four participation statuses were assigned at Phases 7, 9 and 11. For the first missingness mechanism (missing cognitive function and smoking status), a probability, *p*_*i*_, of non-response at Phase *t= 3* was generated to be ignorable conditional on age, occupational grade and education, by choosing values for *β*_*0*_*, β*_*1*_*, β*_*2*_ and *β*_*3*_ so the proportion with non-response status was the same as in the Whitehall II study data:

3 $$ logit\left({p}_i\right)={\beta}_0+{\beta}_1{age}_i+{\beta}_2{occupation}_i+{\beta}_3{education}_i $$

From exploring the associations between participation statuses at adjacent phases, we found participants with non-response status at the phase before were more likely to non-respond at the next phase, and participants with response status at the phase before were more likely to respond at the next phase. Therefore, the probability of non-response at Phase 5 was generated separately for response and non-response status at Phase 3, using Eq. 3.

At Phase *t=7*, separately for response and non-response status at Phase 5, a probability of each participation status (s= response, non-response, death or attrition) was generated to be ignorable, conditional on age, occupational grade and education, again choosing values for *β*_*0ts*_*, β*_*1ts*_*, β*_*2ts*_ and *β*_*3ts*_ so the proportion with each participation status was the same as Whitehall II study data:

4 $$ logit\left({p}_{its}\right)={\beta}_{0 ts}+{\beta}_{1 ts}{age}_i+{\beta}_{2 ts}{occupation}_i+{\beta}_{3 ts}{education}_i $$

Any participants with died or attrition status at Phase 7 were assigned these statuses at later phases because, by definition, they do not return to the study (a monotone missingness pattern). This approach using Eq. 4 was repeated at Phases 9 and 11. Some participants status alternates between response and non-response; a non-monotone missingness pattern. Some missing values were also assigned to participants with response status at each phase with an ignorable missingness mechanism conditional on age (item non-response).

Next, the mean cognitive function score for participants with attrition status at Phases 7, 9 and 11 was examined. Currently, attrition status was generated with an ignorable missingness mechanism. To create a non-ignorable missingness mechanism, the probability of attrition *p*_*i,j*_ was generated by conditioning on the cognitive function values at the same phase *y*_*i,j*_:

5 $$ logit\left({p}_{i,j}\right)={\lambda}_{m\kern0.1em 0}+{\lambda}_{m\kern0.1em 1}{Y}_{i,j} $$

Values for *λ*_*m0*_ and *λ*_*m1*_ were chosen so that the mean cognitive function scores were 0.5 less than the mean scores for an ignorable missingness mechanism, but ensured the proportion of participants with attrition status remained similar to the proportion observed in the Whitehall II study. Using this approach, *k*, from Eq. 2 was assigned the value -0.5. For the first missingness mechanism (missing cognitive function and missing smoking status), we changed cognitive function and smoking status values to missing for participants assigned attrition status at Phases 7, 9 and 11.

For the second missingness mechanism (missing cognitive function and missing education) the same method described above was used, except, to ensure an ignorable missingness mechanism, smoking status at baseline, instead of education, was conditioned on in Eqs. 3 and 4.

As a sensitivity analysis, the effect of increasing the percentage of participants with non-ignorable missingness mechanism was investigated by changing non-response and attrition status to non-ignorable missing at Phases 7, 9 and 11.

In summary, PMM in eight different settings was investigated, defined by the following criteria:

- Outcome.

- (i) Global cognitive function.
- (ii) Memory cognitive function.

b. Missing mechanism.

- (i) Cognitive function and smoking status.
- (ii) Cognitive function and education.

c. Groups assigned missing values using non-ignorable missingness mechanism.

- (i) Attrition.
- (ii) Attrition or non-response.

### Statistics used in the evaluation

Let ${\widehat{\theta}}_m$ denote the parameter estimate for each simulation *m = 1,...,M*. From Rubin’s conditions for proper imputation [2], ${\widehat{\theta}}_m$ is normally distributed with mean *θ* and variance *σ*^*2*^. For *θ*, the true parameter value used in the data generation mechanism, the following statistics were calculated:

1. *Bias(*$\widehat{\theta}$*;θ),* the average of the difference between simulated mean and ‘true’ parameter across the simulations
  $$ \frac{1}{M}\sum \limits_{m=1}^M\left({\widehat{\theta}}_m-\theta \right) $$
2. Empirical variance *Var(*$\widehat{\theta}$*):*
  $$ \frac{1}{M-1}\sum \limits_{m=1}^M{\left({\hat{\theta}}_m-\overline{\hat{\theta}}\right)}^2 $$

where the average imputed mean across simulations is given by:

$$ \overline{\widehat{\theta}}=\frac{1}{M}\sum \limits_{m=1}^M{\widehat{\theta}}_m $$

Smaller variance suggests greater precision (more accurate result).

3. Mean square error (MSE)
  $$ MSE\left(\widehat{\theta}\right)= Var\left(\widehat{\theta}\right)+ Bias{\left(\widehat{\theta},\theta \right)}^2 $$

Smaller MSE suggest less bias. We calculate a ratio of each MSE and the AC analysis MSE for comparison.

4. Confidence interval coverage [25], i.e. the proportion of the *M* confidence intervals
  $$ {\widehat{\theta}}_m\pm {t}_{\delta_m,0.975}\sqrt{{\widehat{\sigma}}_m^2} $$

that include the true value, *θ*. *δ*_*m*_ is the degrees of freedom calculated using Rubin's rules. A 95% level of confidence was used, so 95% of the confidence intervals were expected to contain *θ*.

To aid understanding of the results, we also assessed the correlations between variables in the simulated data, the data with missing values, the data imputed using the two-fold FCS algorithm and the imputed data adjusted using PMM. We performed the analysis using Stata 14 (StatCorp LP, Texas, USA) (www.stata.com) and the two-fold FCS algorithm using the Stata command twofold [26].

## Results

Table 1 shows the characteristics of the participants in the simulated dataset at Phases 5, 7, and 9. The greatest proportions of participants came from the two younger age categories, highest employment grades and education categories. Due to the study design, 49.4% of participants were never smokers at all phases, while the percentage of smokers decreased between Phase 5 (7.2%) and Phase 9 (5.0%). The global and memory cognitive function scores (standardised using mean and SD from Phase 5) decreased between Phase 5 and Phase 9 by 0.42SD and by 0.26SD respectively. The standardised SD for both cognitive function scores was 1 in the Whitehall II study cohort used to generate the simulated data, but less than 1 in the simulated data. Most responded at each phase but attrition (informed Whitehall II they no longer wished to participate) increased from 4.3% at Phase 7 to 6.0% at Phase 9. It was assumed that those participants (approximately 5%) with missing data due to attrition were informative. Approximately 17% were missing due to non-response or death and it was assumed that these were non-informative. In total, approximately 22% of participants had missing values. The analysis was repeated assuming missing due to attrition or non-response were informative, but the results were not reported here.

**Table 1:** Characteristics of participants in each simulated dataset

Phase	5	7	9
Smoking status, n (%)			
Non-smoker	4940 (49.4)	4940 (49.4)	4940 (49.4)
Ex-smoker	4343 (43.4)	4436 (44.4)	4557 (45.6)
Current smoker	717 (7.2)	624 (6.2)	503 (5.0)
Age Category (year), n (%)			
< 50	2420 (24.2)		
50 and < 55	2967 (29.7)		
55 and < 60	2010 (20.1)		
60 and < 65	1896 (19.0)		
65	707 (7.1)		
Employment Grade, n (%)			
High	5812 (58.1)		
Intermediate	3878 (38.8)		
Low	310 (3.1)		
Education, n (%)			
None	555 (5.6)		
School	4675 (46.8)		
University	4770 (47.7)		
Standardised cognitive function (SD), mean (SD)			
Global	0.00 (0.78)	−0.22 (0.78)	− 0.42 (0.79)
Memory	0.05 (0.71)	− 0.10 (0.71)	− 0.26 (0.72)
Participation status, n (%)			
Response	8709 (87.1)	7850 (78.5)	7549 (75.5)
Died	0	240 (2.4)	560 (5.6)
Non-response	1291 (12.9)	1485 (14.9)	1289 (12.9)
Attrition	0	425 (4.3)	602 (6.0)

The bias and precision of the intercept coefficients were similar across the three estimation methods and we have, therefore, restricted our description to the slope coefficients and SE from the mixed effects models. High correlations among repeated global cognitive measures (≈ 0.97) and repeated smoking status measures in the simulated data (≈ 0.95) were observed (Table 2).

**Table 2:** Correlations among variables in full simulated global cognitive function data and differences compared to correlations among variables in available case analyses data, data imputed using multiple imputation and after applying pattern mixture modelling

		Global cognitive function				Smoking status		Age	Grade	Education
	Phase	5	7	9	5	7	9	5	5	5
Full simulated data	Global 5	1								
	Global 7	0.9686	1							
	Global 9	0.9629	0.9691	1						
	Smoke 5	−0.0721	− 0.0860	− 0.0978	1					
	Smoke 7	−0.0595	− 0.0721	− 0.0824	0.9561	1				
	Smoke 9	−0.0467	− 0.0587	− 0.0693	0.9233	0.9537	1			
	Age	−0.2280	− 0.2780	− 0.3293	0.0356	0.0440	0.0538	1		
	Grade	−0.4746	− 0.4462	− 0.4203	0.1134	0.0985	0.0905	−0.0516	1	
	Education	0.3794	0.3721	0.3664	−0.1144	− 0.1056	− 0.1053	− 0.0782	− 0.3666	1
Differences^a^ in correlations from those above										
Available case	Global 5	0								
	Global 7	0.0055	0							
	Global 9	0.0025	0.0067	0						
	Smoke 5	−0.0051	0.0035	0.0062	0					
	Smoke 7	− 0.0058	0.0011	0.0019	−0.0047	0				
	Smoke 9	−0.0106	− 0.0022	− 0.0025	− 0.0051	−0.0011	0			
	Age	−0.1419	−0.1809	− 0.2112	−0.0332	− 0.0283	−0.0265	0		
	Grade	−0.0142	− 0.0036	− 0.0020	0.0107	0.0099	0.0086	−0.0040	0	
	Education	0.0503	0.0535	0.0446	−0.0162	− 0.01520	− 0.0133	− 0.0351	−0.0342	0
Multiple imputation	Global 5	0								
	Global 7	0.0028	0							
	Global 9	0.0198	0.0070	0						
	Smoke 5	0.0463	0.0485	0.0593	0					
	Smoke 7	0.0481	0.0505	0.0631	0.0073	0				
	Smoke 9	0.0389	0.0436	0.0564	0.0130	0.0076	0			
	Age	−0.0160	−0.0205	0.0013	−0.1233	− 0.1276	− 0.1280	0		
	Grade	0.0078	0.0141	0.0063	−0.0173	− 0.0173	− 0.0122	0	0	
	Education	0.0009	−0.0010	−0.0008	0.0280	0.0260	0.0298	0	0	0
Pattern mixture modelling	Global 5	0								
	Global 7	0.0102	0							
	Global 9	0.0275	0.0076	0						
	Smoke 5	0.0463	0.0568	0.0679	0					
	Smoke 7	0.0481	0.0581	0.0710	0.0073	0				
	Smoke 9	0.0389	0.0511	0.0637	0.0130	0.0076	0			
	Age	−0.0160	− 0.0190	− 0.0021	− 0.1233	− 0.1276	− 0.1280	0		
	Grade	0.0078	0.0173	0.0112	−0.0173	− 0.0173	− 0.0122	0	0	
	Education	0.0009	−0.0035	−0.0038	0.0280	0.0260	0.0298	0	0	0

^a^Differences in correlations are calculated as correlation in analysis type minus correlation in full simulated data

The correlations between repeated global cognitive measures and smoking status measures ranged from -0.0467 to -0.0978 and correlations between other variables had similar low correlations (Table 2). The mixed effects substantive model was fitted to each full simulated dataset and the slope coefficients and SE averaged to estimate global cognitive function change over time. The slope coefficients from the full simulated data analysis closely replicated the slope coefficients observed in the Whitehall II study, and were precise due to high correlations among repeated global cognitive function measures (Table 3).

**Table 3:** Slope coefficients and SE from mixed effects substantive model (random intercepts and slopes) with global cognitive score outcome and 5% of participants with informative attrition

	Observed in Whitehall II study	Full simulated data	Estimation method			Estimation method		
			Available case	MI	PMM	Available case	MI	PMM
				Impute smoking^a^			Impute education^a^	
Coefficient (SE)								
Reference	− 0.3120	− 0.3120 (0.0095)	− 0.3082 (0.0139)	−0.3026 (0.0141)	− 0.3401 (0.0153)	− 0.3090 0.0133)	− 0.2885 (0.0104)	− 0.3240 (0.0119)
Smoking status								
Ex-smoker	−0.3271	− 0.3272 (0.0096)	− 0.3236 (0.0142)	− 0.3208 (0.0145)	− 0.3710 (0.0159)	− 0.3244 (0.0135)	− 0.3040 (0.0108)	− 0.3377 (0.0123)
Current smoker	−0.4228	− 0.4229 (0.0110)	− 0.4185 (0.0159)	− 0.3970 (0.0162)	− 0.4558 (0.0176)	−0.4190 (0.0156)	− 0.3975 (0.0131)	−0.4447 (0.0152)
Age Category (year)								
50 and < 55	−0.3619	−0.3619 (0.0091)	− 0.3577 (0.0135)	−0.3510 (0.0138)	− 0.3925 (0.0151)	−0.3585 (0.0129)	− 0.3377 (0.0100)	−0.3779 (0.0117)
55 and < 60	− 0.4400	−0.4398 (0.0093)	−0.4350 (0.0139)	− 0.4262 (0.0142)	− 0.4789 (0.0156)	− 0.4359 (0.0132)	− 0.4133 (0.0105)	− 0.4644 (0.0124)
60 and < 65	− 0.5029	−0.5029 (0.0095)	−0.4971 (0.0154)	− 0.4824 (0.0158)	− 0.5517 (0.0170)	− 0.4984 (0.0148)	− 0.4700 (0.0128)	− 0.5390 (0.0145)
65	−0.5699	− 0.5703 (0.0108)	−0.5648 (0.0419)	− 0.5382 (0.0417)	−0.5717 (0.0425)	− 0.5637 (0.0433)	−0.5204 (0.0433)	− 0.5588 (0.0438)
Employment grade								
Intermediate	−0.2481	−0.2483 (0.0091)	−0.2434 (0.0135)	− 0.2358 (0.0137)	− 0.2928 (0.0151)	− 0.2443 (0.0126)	− 0.2237 (0.0100)	− 0.2805 (0.0117)
Low	− 0.2178	− 0.2173 (0.0129)	− 0.2093 (0.0198)	− 0.1974 (0.0200)	− 0.2963 (0.0226)	−0.2096 (0.0189)	− 0.1857 (0.0178)	−0.2870 (0.0208)
Education								
School	−0.3222	−0.3223 (0.0052)	− 0.3214 (0.0063)	−0.3198 (0.0064)	− 0.3260 (0.0068)	−0.3213 (0.0059)	− 0.3165 (0.0059)	−0.3274 (0.0063)
University	−0.3232	− 0.3232 (0.0043)	− 0.3229 (0.0051)	− 0.3224 (0.0052)	− 0.3220 (0.0055)	− 0.3229 (0.0052)	− 0.3241 (0.0051)	− 0.3304 (0.0054)

*MI* multiple imputation, *PMM* pattern mixture modelling

^a^Missing global cognitive function scores also imputed

**Fig. 1:**
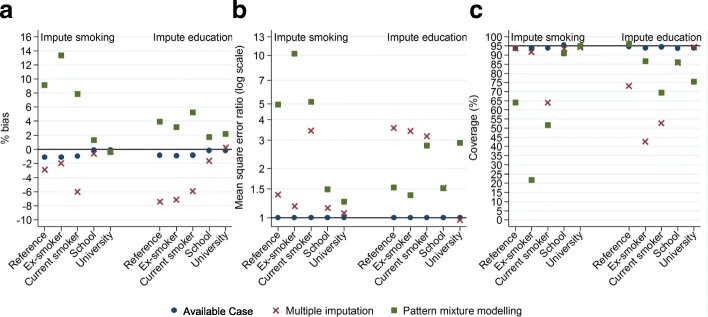
Slope coefficient bias for smoking status and education categories with global cognitive function outcome when imputing the outcome together with either smoking status or education. **a** - % bias, **b** - mean square error ratio, **c** – coverage. **a** - % bias closest to zero indicates least bias approach. **b** - Ratios relative to available case analysis MSE. Ratio less than one indicates less bias compared to the available case analysis. **c** - Coverage close to 95% indicate proper control of the type I error rate for testing a null hypothesis of no effect

After global cognitive function and smoking status were replaced with missing values, the slope coefficients were slightly underestimated when the mixed effects model was fitted to the AC, had greater underestimation when analysing data imputed using the two-fold FCS algorithm and showed overestimation when analysing imputed data adjusted using PMM (Table 3). The slope coefficients from the AC were less precise compared to full simulated data, but more precise compared to fitting the mixed model to data imputed using the two-fold FCS algorithm or imputed data adjusted using PMM (Table 3). Figure 1 shows the bias, MSE and coverage of the different methods and confirms that fitting the mixed effects model to the AC achieved the least biased results.

Again, the coefficients from fitting the mixed effects model to the AC when global cognitive function and education are missing were similar, but less precise, compared to the coefficients from the full simulated data analysis (Table 3). All slope coefficients from fitting the mixed effects model to data imputed using the two-fold FCS algorithm were similar but more precise compared to the AC with education missing, but the slope coefficients were less precise than when smoking status was missing for both variables with and without missing data. With global cognitive function and education missing, less bias was again observed in the slope coefficients from fitting the mixed effects model to the AC compared to data imputed using the two-fold FCS algorithm or imputed data adjusted using PMM (Fig. 1). The AC coefficients and precision were similar in both analyses with smoking status or education missing. However, % bias (Fig. 1a) and MSE (Fig. 1b) were smaller and coverage was closer to 95% (Fig. 1c) for the slope coefficients from fitting the mixed effects model to the AC compared to analysing data imputed using the two-fold FCS algorithm or imputed data adjusted using PMM (Fig. 1).

The correlations among repeated memory cognitive function were approximately 0.45, less than half of those observed for global cognitive function (Table 4). Correlations among repeated smoking status measurements had similar high correlations to the global cognitive function data (Table 2). However, correlations between all other covariates were low (Table 4). With missing memory cognitive function and smoking status, the mixed effects model fitted to the AC, gave larger underestimated slope coefficients and were less precise (Table 5) compared to those with global cognitive function, due to lower correlations among the repeated memory measures. The slope coefficients from fitting the mixed effects model to data imputed using the two-fold FCS algorithm were generally more underestimated, but also more precise, compared to AC (Table 5) due to higher correlations in the imputed data (Table 4). However, the slope coefficients from fitting the mixed effects model to imputed data adjusted using

**Table 4:** Correlations among variables in full simulated memory cognitive score data and differences compared to correlations among variables in available case analyses data, data imputed using multiple imputation and after applying pattern mixture modelling

		Memory cognitive function			Smoking status			Age	Grade	Education
	Phase	5	7	9	5	7	9	5	5	5
Full simulated data	Mem 5	1								
	Mem 7	0.4419	1							
	Mem 9	0.4423	0.4458	1						
	Smoke 5	−0.0806	−0.0884	−0.1041	1					
	Smoke 7	− 0.0715	− 0.0800	− 0.0948	0.9616	1				
	Smoke 9	−0.0636	−0.0764	− 0.0902	0.9337	0.9592	1			
	Age	−0.2729	− 0.3127	− 0.3379	0.0281	0.0369	0.0451	1		
	Grade	−0.2404	− 0.1913	− 0.1660	0.0983	0.0881	0.0817	−0.0501	1	
	Education	0.2058	0.1919	0.1779	−0.1114	− 0.1043	− 0.1005	− 0.0784	− 0.3691	1
Differences^a^ in correlations from those above										
Available case	Mem 5	0								
	Mem 7	0.0355	0							
	Mem 9	0.0544	0.0630	0						
	Smoke 5	0.0159	0.0029	0.0040	0					
	Smoke 7	0.0071	0.0032	0.0004	0.0009	0				
	Smoke 9	0.0137	−0.0005	0.0050	0.0025	0.0087	0			
	Age	−0.1632	− 0.1679	− 0.2154	− 0.0253	− 0.0237	− 0.0194	0		
	Grade	−0.0120	− 0.0102	− 0.0125	0.0066	0.0043	0.0004	−0.0104	0	
	Education	0.0052	0.0032	0.0339	−0.0050	− 0.0022	0.0015	− 0.0151	− 0.0031	0
Multiple imputation	Mem 5	0								
	Mem 7	0.0157	0							
	Mem 9	0.0970	0.0364	0						
	Smoke 5	0.0528	0.0579	0.0306	0					
	Smoke 7	0.0522	0.0550	0.0360	0.0062	0				
	Smoke 9	0.0532	0.0506	0.0377	0.0083	0.0080	0			
	Age	−0.0186	−0.0298	− 0.0269	− 0.1200	− 0.1246	− 0.1237	0		
	Grade	0.0067	− 0.0012	− 0.0070	− 0.0116	− 0.0145	− 0.0079	0	0	
	Education	− 0.0067	0.0169	0.0066	0.0282	0.0272	0.0329	0	0	0
Pattern mixture modelling	Mem 5	0								
	Mem 7	0.0115	0							
	Mem 9	0.0877	0.0099	0						
	Smoke 5	0.0528	0.0675	0.0410	0					
	Smoke 7	0.0522	0.0655	0.0464	0.0062	0				
	Smoke 9	0.0532	0.0612	0.0481	0.0083	0.0080	0			
	Age	−0.0186	− 0.0204	− 0.0194	− 0.1200	− 0.1246	− 0.1237	0		
	Grade	0.0067	0.0020	0.0007	−0.0116	− 0.0145	− 0.0079	0	0	
	Education	−0.0067	0.0123	−0.0001	0.0282	0.0272	0.0329	0	0	0

^a^Differences in correlations are calculated as correlation in analysis type minus correlation in full simulated data

**Table 5:** Slope coefficients and SE from mixed effects substantive model (random intercepts and slopes) with memory cognitive score outcome and 5% of participants with informative attrition

	Observed in Whitehall II study	Full simulated data	Estimation method			Estimation method		
			Available case	MI	PMM	Available case	MI	PMM
				Impute^a^ smoking			Impute^a^ education	
Coefficient (SE)								
Reference	− 0.2492	− 0.2508 (0.0356)	− 0.2320 (0.0507)	− 0.2290 (0.0487)	−0.2439 (0.0486)	− 0.2331 (0.0491)	− 0.1583 (0.0413)	− 0.1765 (0.0414)
Smoking status								
Ex-smoker	−0.2978	−0.2989 (0.0361)	−0.2792 (0.0513)	− 0.2798 (0.0489)	−0.3104 (0.0489)	− 0.2799 (0.0496)	−0.2055 (0.0414)	− 0.2246 (0.0415)
Current smoker	−0.2698	−0.2718 (0.0418)	−0.2459 (0.0581)	− 0.2202 (0.0543)	−0.2564 (0.0544)	− 0.2444 (0.0574)	−0.1724 (0.0474)	− 0.2008 (0.0473)
Age Category (year)								
50 and < 55	−0.3059	−0.3064 (0.0347)	−0.2821 (0.0493)	− 0.2776 (0.0476)	−0.2980 (0.0477)	− 0.2829 (0.0472)	−0.2099 (0.0401)	− 0.2343 (0.0401)
55 and < 60	−0.3405	−0.3414 (0.0357)	−0.3076 (0.0511)	− 0.3003 (0.0492)	−0.3316 (0.0492)	− 0.3085 (0.0489)	−0.2338 (0.0411)	− 0.2687 (0.0410)
60 and < 65	−0.3967	−0.3978 (0.0360)	−0.3486 (0.0553)	− 0.3302 (0.0523)	−0.3831 (0.0526)	− 0.3508 (0.0527)	−0.2661 (0.0456)	− 0.3218 (0.0457)
65	−0.3318	−0.3327 (0.0410)	−0.2803 (0.1437)	− 0.2400 (0.1081)	−0.2561 (0.1078)	− 0.2764 (0.1481)	−0.1795 (0.1132)	− 0.2037 (0.1135)
Employment grade								
Intermediate	-0.1616	− 0.1638 (0.0335)	− 0.1388 (0.0479)	− 0.1342(0.0465)	− 0.1577 (0.0463)	− 0.1390 (0.0462)	− 0.0746 (0.0389)	− 0.1027 (0.0393)
Low	−0.2059	− 0.2080 (0.0484)	− 0.1703 (0.0695)	− 0.1656 (0.0664)	− 0.2031 (0.0659)	− 0.1736 (0.0662)	− 0.1182 (0.0611)	− 0.1602 (0.0617)
Education								
School	−0.2413	− 0.2414 (0.0206)	− 0.2317 (0.0249)	− 0.2298 (0.0238)	− 0.2368 (0.0238)	− 0.2321 (0.0253)	− 0.2116 (0.0233)	− 0.2245 (0.0233)
University	− 0.2533	− 0.2540 (0.0178)	− 0.2480 (0.0212)	− 0.2482 (0.0203)	− 0.2517 (0.0204)	− 0.2480 (0.0211)	− 0.2558 (0.0200)	− 0.2657 (0.0201)

*MI* multiple imputation, *PMM* pattern mixture modelling

^a^Missing memory cognitive function scores also imputed

**Fig. 2:**
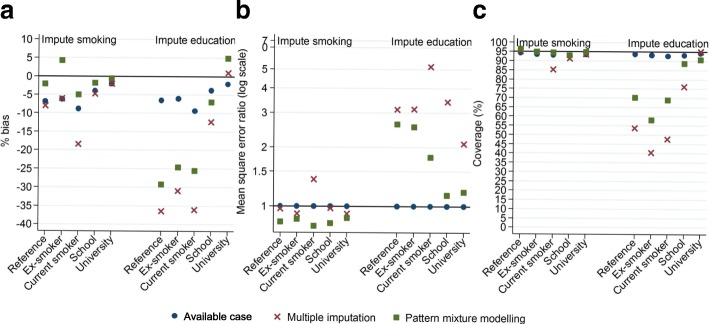
Slope coefficient bias for smoking status and education categories with memory cognitive function outcome when imputing the outcome together with either smoking status or education. **a** - % bias, **b** - mean square error ratio, **c** – coverage. **a** - % bias closest to zero indicates least bias approach. **b** - Ratios relative to available case analysis MSE. Ratio less than one indicates less bias compared to the available case analysis. **c** - Coverage close to 95% indicate proper control of the type I error rate for testing a null hypothesis of no effect

PMM were similar to the full data analysis coefficients compared to fitting mixed effects model to the AC or data imputed using the two-fold FCS algorithm (Table 5). Figure 2a confirms that slope coefficients from analysing imputed data adjusted using PMM had the least bias, smallest MSE (Fig. 2b) and coverages closest to 95% (Fig. 2c). Using the two-fold FCS algorithm to impute the missing values increased precision, but some bias still existed in the coefficients, which was reduced, but not completely removed, using PMM (Fig. 2).

When memory cognitive function and education were missing, more precise slope coefficients but greater underestimation was observed when fitting the mixed effects model to data imputed using the two-fold FCS algorithm compared to missing memory cognitive function and smoking status since we did not condition on repeated education measurements at other phases to reduce bias. Due to the larger bias in the coefficients from analysing data imputed using the two-fold FCS algorithm, adjusting the imputed data using PMM did not reduce this to less than the AC analysis slope coefficients, which had least bias (Fig. 2a), smallest MSE (Fig. 2b) and coverages closest to 95% (Fig. 2c).

## Discussion

This study described a PMM approach to account for bias due to informative attrition in a longitudinal, cohort study with non-monotone missing data. We found adjusting imputed data using PMM unnecessary when using a mixed effects substantive model and data with highly correlated repeated outcome measurements. The mixed effects model fitted to AC gave least bias slope coefficient because enough information was available in the repeated measurements.

The mixed effects model slope coefficients from the AC had more bias and less precision with lower to moderate correlations among the repeated outcome measurements, because not enough information is available in the observed data to adequately use the between and within participant correlations to adjust for the missing values. The two-fold FCS algorithm included additional information, not available in the AC, to reduce bias and precision. From fitting the mixed effects model to data imputed using the two-fold FCS algorithm, the time-dependent smoking status explanatory variable slope coefficient increased precision because the two-fold FCS algorithm conditions on highly correlated smoking status measurements at other phases. However, using the two-fold FCS algorithm to impute missing values for the time-independent explanatory variable education did not increase precision as much for variables with and without missing data possibly because no highly correlated repeated measurements exist to condition on. We may have observed greater bias reduction since education had a higher correlation with the outcome compared to smoking status [27]. Some bias in the slope coefficients from analysing data imputed using the two-fold FCS algorithm was still observed, but this reduced after adjusting the imputed data using PMM. However, the slope coefficients from fitting the mixed effects model to imputed data adjusted using PMM still underestimated the full data analysis slope coefficients. Although it is unlikely that such high correlations, as seen among repeated global cognitive function, will be observed in existing epidemiological datasets, it is more likely that correlations like those seen for repeated memory cognitive function will be observed. However, the results from analysing data with high and moderate correlations among repeated outcome measurements can be compared.

The intercept coefficients were not reported in the results since fitting the mixed effect model to the AC and data imputed using the two-fold FCS algorithm showed similar bias and precision. For some analyses, for example with the global cognitive function outcome, slightly more precise and less bias intercept coefficients were observed when analysing data imputed using the two-fold FCS algorithm compared to AC analysis, but the AC analysis achieved the least bias slope estimates. However, the difference in bias between analysing the AC and imputed data was small, and it may be preferable to analyse the AC in practice. For memory cognitive function outcome, the least bias intercept and slope coefficients were observed from fitting mixed effects model to imputed data adjusted using PMM.

Some participants chose to stop participation in the Whitehall II study, but did not formally withdraw, so may contribute to the bias due to informative attrition. Initially, it was assumed that all participants who formally withdrew were due to informative attrition. The analyses were repeated, overestimating the bias by assuming all participants with attrition and non-response status contributed to the bias due to informative attrition, which increased the percentage with non-ignorable missingness from 5 to 20%. The coefficients had larger bias compared to 5%, but the general findings were the same (results not shown).

Historically, the literature recommended using MI to impute missing values and then delete imputed outcome values before analysis. If both outcome and explanatory variables have missing values, imputing both outcome and explanatory variables will provide some information for the substantive model, by improving prediction of missing explanatory variables with observed outcome [14], but cases with imputed outcome contain no information about the regression of the outcome on explanatory variables [28]. However, more recent research does not recommended deletion since analysing data imputed using an imputation model with auxiliary variables associated with missing outcome found biased coefficients when observations with imputed outcomes were removed from the analysis [29]. We therefore chose to use MI to impute all the missing values and analyse the imputed data without any deletion.

For this paper, auxiliary variables were not included in the imputation model to simplify the analysis and interpretation. Slope coefficients from fitting mixed effects model to imputed data adjusted using PMM for moderately correlated time-dependent outcomes were underestimated. To increase precision, auxiliary variables highly correlated with the outcome values could be included, and this also reduces bias if the auxiliary variables are also correlated with the probability the variable is missing [30]. A monotone observational study investigated MI and a joint model of the cross-sectional and longitudinal data [31]. Under non-ignorable missingness, both methods resulted in biased estimates. However, including auxiliary variables correlated with the variables with missing values reduced the bias. Wang, et al., recommend future work to evaluate the effectiveness of auxiliary variables to impute missing values in non-monotone missingness data [31].

A prospective cohort study investigated the association between diabetes diagnosis and cognitive decline using a mixed effects model (which implicitly assumes the same distribution for those who drop out and for those who stay in the study) and used generalised estimating equations to avoid the implicit imputation [32]. In the study, they imputed missing scores due to drop out for both alive and deceased and investigated the effect of including auxiliary variables associated with cognitive function and the probability of drop out and death. Rawlings, et al., found similar results for mixed effects model and generalised estimating equations [32]. However, when auxiliary variables associated with drop out were included in the imputation model, MI effectively reduced the bias in the estimates. Some clinical trial studies with monotone missing data have also investigated incorporating reason for drop out. Standard PMM assumes non-random drop out and Lotz, et al., found that, when additionally stratifying standard PMM by random and non-random reasons for drop-out, the results had less bias [33]. For the purposes of this simulation study, a simple missingness mechanism was chosen where the outcome was associated with drop out. However, in reality, it is likely to be more complex and associated with other variables such as smoking status. Mein, et al., investigated risk factors associated with drop out in the Whitehall II study, which could be incorporated in the analysis [34].

Biering, et al., investigated a similar MI approach to the two-fold FCS algorithm when imputing missing values in a non-monotone missing longitudinal observational data; the Mental Component Summary from Short Form 12-item survey [35]. This study imputed missing data due to non-response and attrition at each time conditional on measurements at adjacent time and death, but did not impute measurements missing due to death. Biering, et al., changed imputed outcome values by a fixed amount, similar to PMM, which also effectively accounted for a non-ignorable missingness mechanism.

The main strength of this study was using a large, complex cohort in a real-life epidemiological setting to describe PMM in non-monotone missing cohort data; the findings are likely to be generalisable to other longitudinal studies. Using the two-fold FCS algorithm is another strength because it is the appropriate approach for imputing non-monotone longitudinal data, particularly for time-dependent variables, which imputes missing values for each phase sequentially conditional on observed information at adjacent phases [4]. This approach avoids possible convergence problems due to collinearity by restricting imputation to a short time window. Also, the results from a simulation study found increased precision when analysing time-dependent explanatory variables imputed using the two-fold FCS algorithm compared to standard approaches, such as AC [5]. The two-fold FCS algorithm can reduce bias and increase precision by conditioning on correlated repeated measures of the time-dependent outcome variable at adjacent phases. Therefore, using PMM to adjust data imputed using the two-fold FCS algorithm may be most suitable for longitudinal studies with many measurement phases, participants and variables. A previous study that used the two-fold FCS to impute missing outcome and explanatory variables found similar results to other MI approaches, but these analyses were restricted to 3 data collection waves [36].

A limitation of using the two-fold FCS algorithm is that it overestimates the random slope (results not shown) because it does not correctly consider multilevel structure by conditioning on the random intercept and slope in the imputation [37]. However, methods described in this paper are suitable for fixed effects. Demirtas and Shchafer investigated using MI to average marginal estimates from each pattern [8]. The authors observed under coverage in the results because of uncertainty due to model misspecification was not taken into account. However, they repeated the imputations using a three-level linear mixed effects imputation model which included a random level due to each pattern, accommodating model uncertainty in the imputation process [38].

A potential limitation is that *k*, the mean difference between the imputed outcome values and the unknown missing outcome values, was assumed to be constant, and this may be unrealistic [3]. For a more general specification in future studies, a distribution for *k* could be specified and a sensitivity analysis performed to investigate the effects of changing the variance of *k* as well its mean. Also, PMM may not reduce bias for outcome-dependent, non-ignorable missingness if large residual errors exist since the probability of missingness depends on residual errors as well as true outcome values [39]. For instance, participants with high observed outcome scores who are more likely to drop out may also have high measurement error values and, therefore, the mean of measurement errors within each missing pattern may no longer be zero. This may be an issue with the Whitehall II cognitive function data. Participants know of the tests in advance, since the tests repeat at each phase, so participants can prepare [40] and a higher than expected cognitive functioning in participants has been observed. However, in the data simulated for this paper, the residual error for each missing pattern was examined and the means were close to zero.

The National Research Council panel for handling missing data in clinical trials, USA, recom-mended undertaking more research to understand appropriate methods to impute missing values in non-monotone data [41], so this study adds to the evidence base.

## Conclusions

Our findings suggest that with moderate correlations in the repeated outcome measurements and a linear mixed effects substantive model, using PMM reduces bias and increases precision but may still underestimate the true slope coefficient. With high correlations between repeated outcome measurements, the linear mixed effects model fitted to the available cases can adequately recover information. We recommend a few considerations for further analysis when using PMM, which may reduce bias and increase precision. First, select appropriate auxiliary variables for the imputation model with highly correlated repeated measurements or correlated with the outcome. Also, incorporate the reason for drop out in the imputation model.

## Additional file

Additional file 1: Appendix. (DOCX 17 kb)

## Abbreviations

AC: Available cases
CI: Confidence interval
FCS: Fully conditional specification
MI: Multiple imputation
MSE: Mean square error
PMM: Pattern mixture modelling
SE: Standard errors
