# Supplementary material for: Does pattern mixture modelling reduce bias due to informative attrition compared to fitting a mixed effects model to the available cases or data imputed using multiple imputation?: a simulation study
Source: BMC Med Res Methodol. 2018 Aug 29;18:89. doi: 10.1186/s12874-018-0548-0 (PMC6114233; doi:10.1186/s12874-018-0548-0)
Supplement: Supplementary file 1 — Appendix. (DOCX 17 kb) [file 12874_2018_548_MOESM1_ESM.docx]

**Appendix: Data generation mechanism**

We now describe the first data generation mechanism. For each patient *i = 1; : : : ; N* and *t = 1; : : : ; 5*, corresponding to phases 3, 5, 7, 9 and 11:

- Generate baseline occupation grade (at *t = 2*), denote *occupation_i_* with values *a = 1; 2; 3* corresponding to high, intermediate and low occupation grades respectively, from an unadjusted ordinal logistic model;

*logit{Pr(occupation_i_ = a)} =* $\beta_{0,a}^{occu}$

where a = 1 (high - reference category); 2 (intermediate); 3 (low)

- Generate baseline age (at *t = 2*) and denoted age_i_ with values *b = 1; : : : ; 5* corresponding to 5-year age categories, from an ordinal logistic regression model conditional on baseline occupation grade:

*logit{Pr(age_i_ = b)} =* $\beta_{1,b}^{age}+\sum_{a=1}^{3} \beta_{1,b}^{age}\left[ {occupation}_{i}=a \right]$

- Generate highest academic qualification, denoted *education_i_* with values *c = 1; 2; 3*, from an ordinal logistic regression model conditional on baseline occupation grade and baseline age:

*logit{Pr(education_i_ = c)} =* $\beta_{0,c}^{educ}+\sum_{a=1}^{3} \beta_{1,c}^{educ}[oc{cupation}_{i}=a]+ \sum_{b=1}^{5} \beta_{2,c}^{educ}[{age}_{i}=b]$

where c = 1 (no education - reference category); 2 (secondary education); 3 (university education)

- Generate baseline smoking status (at *t = 2*) denoted *smoke_i;t_* with values *d = 1; 2; 3,* from a multinomial logistic model conditional on baseline occupational grade, age category, and highest ever academic qualification:

*logit{Pr(smoke_i,2_ = d)} =* $\beta_{0,d}^{smoke}+\sum_{a=1}^{3} \beta_{1,d}^{smoke}\left[ occupation_{i}=a \right]+$

$\sum_{b=1}^{5} \beta_{2,d}^{smoke}\left[ age_{i}=b \right]+\sum_{c=1}^{3} \beta_{3,d}^{smoke}[education_{i}=c]$

where d = 1 (never smoker - reference category); 2 (ex-smoker); 3 (current smoker); Never-smokers at baseline were indicated as never smokers for each subsequent phase. To generate the remaining smoking status (smokers or ex-smokers) at *t = 3; 4; 5,* we used different probabilities *s_d;t_* dependent on smoking status at previous phase:

$$\Pr\left( smoke_{i,t}=d | smoke_{i,t}\neq1 \right)=\left\{ \begin{aligned} s_{2,t} if smoke_{i,t-1}=2, and \\ s_{3,t} if smoke_{i,t-1}=3 \end{aligned} \right.$$

- We generated global and memory cognitive function outcome cog_i;t_ from a linear mixed effects regression model with interactions between each coefficient and time and random intercept β_0i_ and slope β_1i_:

${cog}_{i,t}=\beta_{0,t}^{cog}+\sum_{a=1}^{3} \beta_{1,t}^{cog}\left[ occupation_{i}=a \right]+ \sum_{a=1}^{3} \beta_{2,t}^{cog}\left( \left[ occupation_{i}=a \right]\times t \right)+$

$\sum_{b=1}^{5} \beta_{3,t}^{cog}\left[ age_{i}=b \right]+\sum_{b=1}^{5} \beta_{4,t}^{cog}\left( \left[ age_{i}=b \right]\times t \right)+$

$\sum_{c=1}^{3} \beta_{5,t}^{cog}\left[ education_{i}=c \right]+\sum_{c=1}^{3} \beta_{6,t}^{cog}\left( \left[ education_{i}=c \right]\times t \right)+$

$\sum_{d=1}^{3} \beta_{7,t}^{cog}\left[ smoke_{i}=d \right]+\sum_{d=1}^{3} \beta_{8,t}^{cog}\left( \left[ smoke_{i}=d \right]\times t \right)+t+ \beta_{0i}+\beta_{1i}t+\varepsilon_{i,t}$
